# Factors Affecting the Referral Time to Nephrologists in Patients With Chronic Kidney Disease: A Prospective Cohort Study in Korea

**DOI:** 10.1097/MD.0000000000003648

**Published:** 2016-05-13

**Authors:** Jeonghwan Lee, Jung Pyo Lee, Jung Nam An, Sung Gyun Kim, Yong-Lim Kim, Chul Woo Yang, Shin-Wook Kang, Nam-Ho Kim, Yon Su Kim, Yun Kuy Oh, Chun Soo Lim, and Clinical Research Center for End Stage Renal Disease (CRC for ESRD) Investigators

**Affiliations:** From the Department of Internal Medicine (JL), Hallym University Hangang Sacred Heart Hospital; Department of Internal Medicine (JPL, JNA, YKO, CSL), Seoul National University Boramae Medical Center, Seoul; Department of Internal Medicine (SGK), Hallym University Sacred Heart Hospital, Anyang; Department of Internal Medicine (Y-LK), Kyungpook National University School of Medicine, Daegu; Department of Internal Medicine (CWY), Seoul St. Mary's Hospital, College of Medicine, The Catholic University of Korea; Department of Internal Medicine (S-WK), Yonsei University College of Medicine, Seoul; Department of Internal Medicine (N-HK), Chonnam National University Medical School, Gwangju; Department of Internal Medicine (YSK), Seoul National University College of Medicine, Seoul; and Clinical Research Center for End Stage Renal Disease (CRC for ESRD), Daegu (JL; JPL; JNA; SGK; Y-LK; CWY; S-WK; N-HK; YSK; YKO; CSL), Republic of Korea.

## Abstract

Timely referral to nephrologists is important for improving clinical outcomes and reducing costs during transition periods. We evaluated the impact of patients’ demographic, clinical, and social health characteristics on referral time.

A total of 1744 CKD patients who started maintaining dialysis were enrolled in a Korean prospective cohort. The early referral (ER) and late referral group (LR) were defined as patients who were referred to a nephrologist more than or less than 1 year prior to dialysis initiation, respectively.

A total of 1088 patients (62.3%) were in the ER, and 656 patients (37.6%) were in the LR. Among the patients in the LR, 398 patients (60.7%) were referred within the 3 months prior to the start of dialysis (ultralate referral group [ULR]). The ER was younger at the time of referral than the LR; however, the ER was older at the start of dialysis. Patients with diabetes or hypertension as the cause of kidney disease were more common in the LR, whereas patients with glomerulonephritis, females, and nonsmokers were more common in the ER. The ER had more well-controlled blood pressure, lower phosphorus levels, and higher hemoglobin levels at the start of dialysis. Congestive heart failure (CHF) was more common in the LR. In the multivariate analysis, male sex (odds ratio [OR] 1.465, 95% confidence interval [CI] 1.034–2.076), underlying kidney disease (diabetes mellitus [OR 1.507, 95% CI 1.057–2.148] and hypertension [OR 1.995, 95% CI 1.305–3.051]), occupation (mechanician [OR 2.975, 95% CI 1.445–6.125], laborer [OR 3.209, 95% CI 1.405–7.327], and farmer [OR 5.147, 95% CI 2.217–11.953]), CHF (OR 2.152, 95% CI 1.543–3.000), and ambulatory status (assisted-walks, OR 2.072, 95% CI 1.381–3.111) were proved as the independent risk factor for late referral.

Patients with hypertensive or diabetic kidney disease are referred later than those with glomerulonephritis. Male patients with physically active occupations exhibiting CHF and restricted ambulation were associated with a late referral. Considering the various factors associated with late referral, efforts to increase early referrals should be emphasized, particularly in patients with hypertension, diabetes, or congestive heart failure.

## Abstract 2

Supplemental Digital Content is available in the text

## INTRODUCTION

In patients with chronic kidney disease (CKD), timely referral to nephrologists and adequate care are important for improving the patients’ clinical outcomes. The benefits of early referral to nephrologists have been well investigated in previous studies. Patients who were referred early showed a reduced use of temporary dialysis catheters, a decreased need for urgent dialysis, a time delay until the initiation of renal replacement therapy, and a higher incidence of peritoneal dialysis or kidney transplantation as an initial modality.^1^ Patients referred early are relatively well managed, even with cardiovascular disease and other comorbidities.^2^ Early referral can improve patients’ survival, nutritional status, and quality of life.^3,4^ In addition, early referral can reduce hospitalization, length of hospital stay, and medical costs. We also investigated that patients who were referred early before the start of dialysis had benefits on overall and cardiovascular survival, medical expenses, and quality of life.^5–7^

Although many clinicians have come to understand the importance of timely referral in patients with CKD, a large proportion of patients with CKD are still referred late relative to the start of dialysis. The referral time and proportion of late referral patients vary widely according to the country and definition of late referral. Previous studies have reported that only 20% to 35% patients are referred late.^8^ In the United States, despite a decreasing pattern of late referrals, 34.7% patients were still referred late in 2006.^9^ In Mexico, over 50% of patients were referred late at 1 month before dialysis initiation.^10^ In a Danish cohort study, 38% of patients were referred less than 16 weeks before the start of renal replacement therapy.^11^

To increase the proportion of patients with early referral, an investigation of the clinical and socio-economic factors affecting referral time is required. Scarce data are available on the factors associated with referral time, especially in Asian countries. In this study, we evaluated the impact of patients’ demographic, clinical, and social health characteristics on referral time.

## METHODS

### Cohort Description

This study was investigated as part of a cohort study (Clinical Research Center for End Stage Renal Disease, CRC ESRD) of patients with ESRD in South Korea. The CRC ESRD is a nationwide multicenter web-based prospective cohort of CKD patients receiving dialysis, and its aim is to analyze the effects of treatment effects on survival, quality of life, and cost effectiveness (clinicaltrial.gov NCT00931970). All of the enrolled patients are adults over 20 years old who started dialysis for ESRD without a kidney transplant scheduled within 3 months. Patient registration began in July 2008, and 31 hospitals are currently participating in the CRC ESRD cohort study in South Korea. The study was approved by the institutional review board at each center. All of the patients provided written consent to participate voluntarily in this study. All clinical investigators observed the Ethics for Medical Research and carried out this study in accordance with the guidelines of the 2008 Declaration of Helsinki.

### Study Participants

From the new patient registry in the CRC ESRD cohort, we enrolled 1744 adult patients who started maintenance dialysis for newly diagnosed ESRD between August, 2008 and January, 2015. Patients with missing data on the dates of initial visits to a nephrologist and start dates of dialysis were excluded in this study. The early referral group (ER) was defined as patients who were referred to a nephrologist more than 1 year prior to dialysis initiation, the late referral group (LR) was defined as patients whose referral time was less than a year prior to dialysis initiation, and the ultralate referral group (ULR) was defined as patients whose referral time was less than 3 months prior to dialysis initiation.

### Clinical Data Acquisition

The information used to analyze the participants was primarily collected from the data server of the CRC ESRD. The clinical, demographic, and socio-economic data had been stored in the online accessible web database. The contents of questionnaire were filled in by trained coordinators through medical chart reviews and direct interviews using a standard format. The questionnaires consisted of data on demographics, occupation, insurance status, marriage status, degree of familial and social support, education status, smoking history, previous medical history, laboratory results, dialysis modality and prescription, comorbidities, and medications. The estimated glomerular filtration rate was calculated by CKD-EPI equations.^12^ The modified Charlson comorbidity index was recorded at the time of dialysis for each patient. The questionnaire items were collected from all patients when they visited dialysis facilities or were admitted to hospitals for the first maintenance dialysis.

### Statistical Analysis

In the descriptive analysis of the demographic and clinical characteristics and laboratory results, continuous variables were expressed as the mean and standard deviation, and categorical variables were described numerically with a percentage. All continuous variables were tested for normality distribution by Kolmogorov–Smirnov methods. Comparisons between the groups based on referral time were performed using a *t* test or ANOVA for continuous variables and Chi-square tests for categorical variables when appropriate. Continuous variables that were not in normality distribution were compared with Mann–Whitney *U* test or Kruskal–Wallis test method. Clinical, demographic, and socio-economic factors that are associated with late and ultralate referral were analyzed by logistic regression method. Statistically significant variables that showed *P* value below 0.2 in the univariate analysis were included in a multivariate analysis based on forward selection methods. IBM SPSS ver. 21.0 was used to compare the demographic and clinical parameters. A *P* value < 0.05 was considered statistically significant.

## RESULTS

### Distribution of Referral Time

**FIGURE 1:**
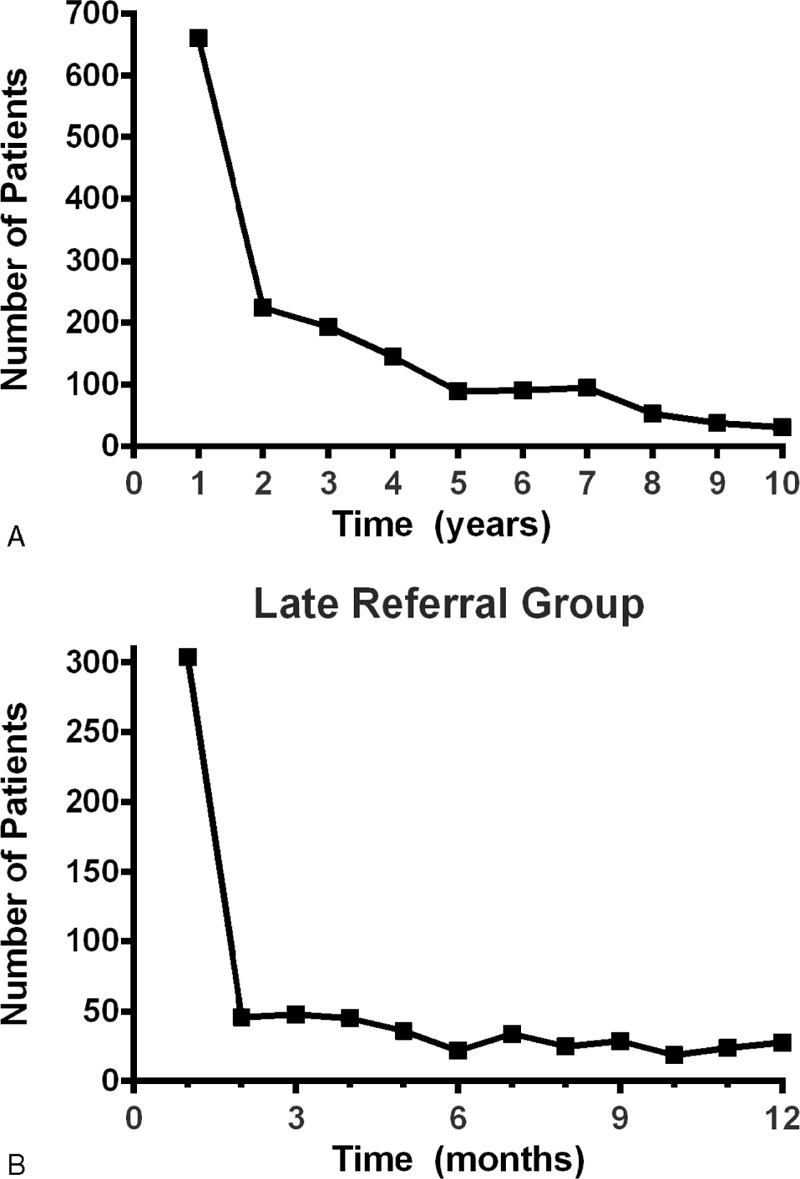
Distribution of referral time from the initial visit to the nephrology clinic to dialysis initiation. (A) Among a total of 1744 study participants, 656 patients (37.6%) were referred late (<1 year before dialysis start) and 1088 patients (62.3%) were referred early (≥1 year before dialysis start). The number of patients who were referred late (short referral time) increased gradually throughout the entire referral time period. (B) Among patients who were referred late to the nephrologist, 398 patients (60.7%) were referred 3 months before dialysis initiation (ultralate referral group) and 304 patients (46.3%) were referred just before dialysis (<1 month before dialysis start).

The patients’ referral times are presented in Figure 1. Among the 1744 study participants, 656 patients (37.6%) were referred late (<1 year before dialysis start) and 1088 patients (62.3%) were referred early (≥1 year before dialysis start) (Figure 1A). The number of patients who were referred late (short referral time) increased gradually throughout the referral time period. Among the 656 patients who were referred late to the nephrologists (<1 year before dialysis start), 398 patients (60.7%) were referred within 3 months prior to starting dialysis (ULR) and 304 patients (46.3%) were referred within 1 month prior to starting dialysis (Figure 1B).

### Patients Characteristics by Referral Pattern

The patients’ clinical and laboratory characteristics relative to the referral time are summarized in Table 1   and Supplementary Table 1. The mean age at the time of referral was 52.8 ± 14.7 years old, and 60.8% of the patients were male. Of the 1744 patients enrolled in this study, 1088 patients were in the ER and 656 were in the LR. The time from referral to dialysis was significantly longer in the ER than in the LR (64.9 ± 58.2 months vs 3.1 ± 3.6 months, *P* < 0.001).

**TABLE 1:**
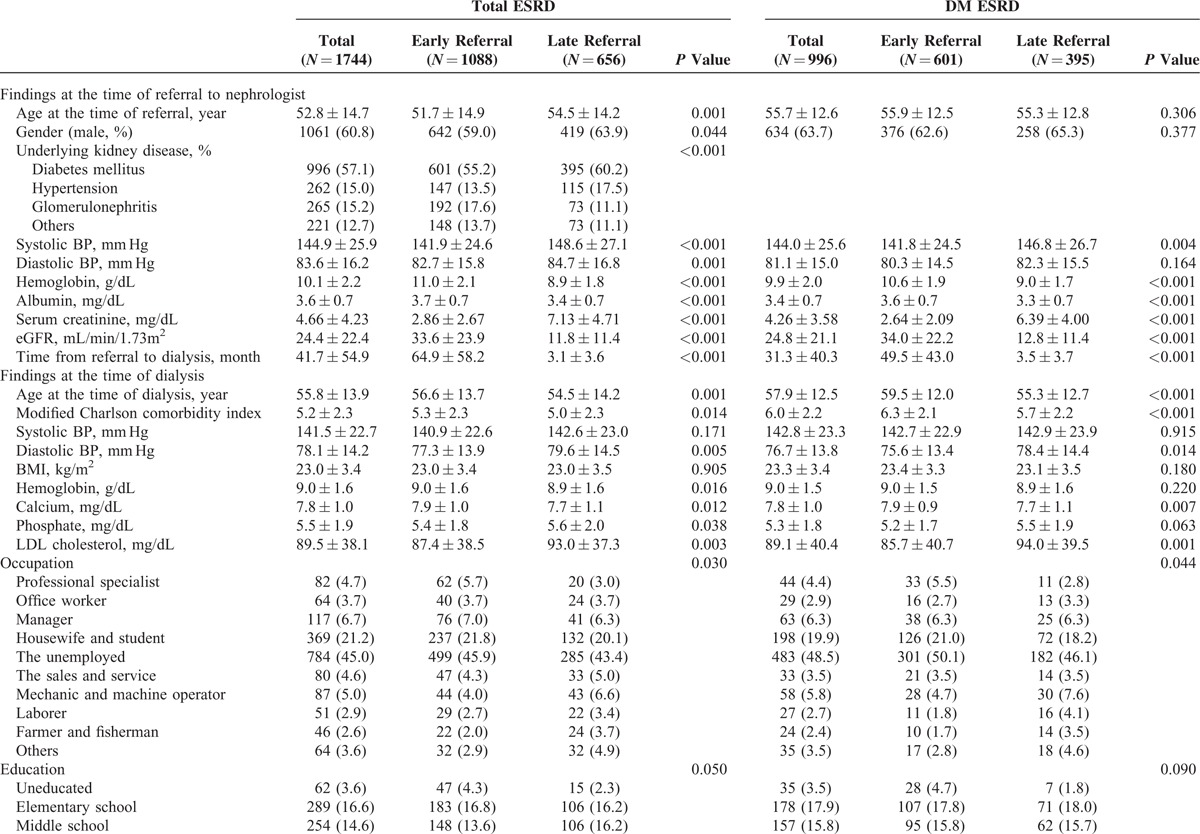
Patient Characteristics According to Referral Time

**TABLE 1 (Continued):**
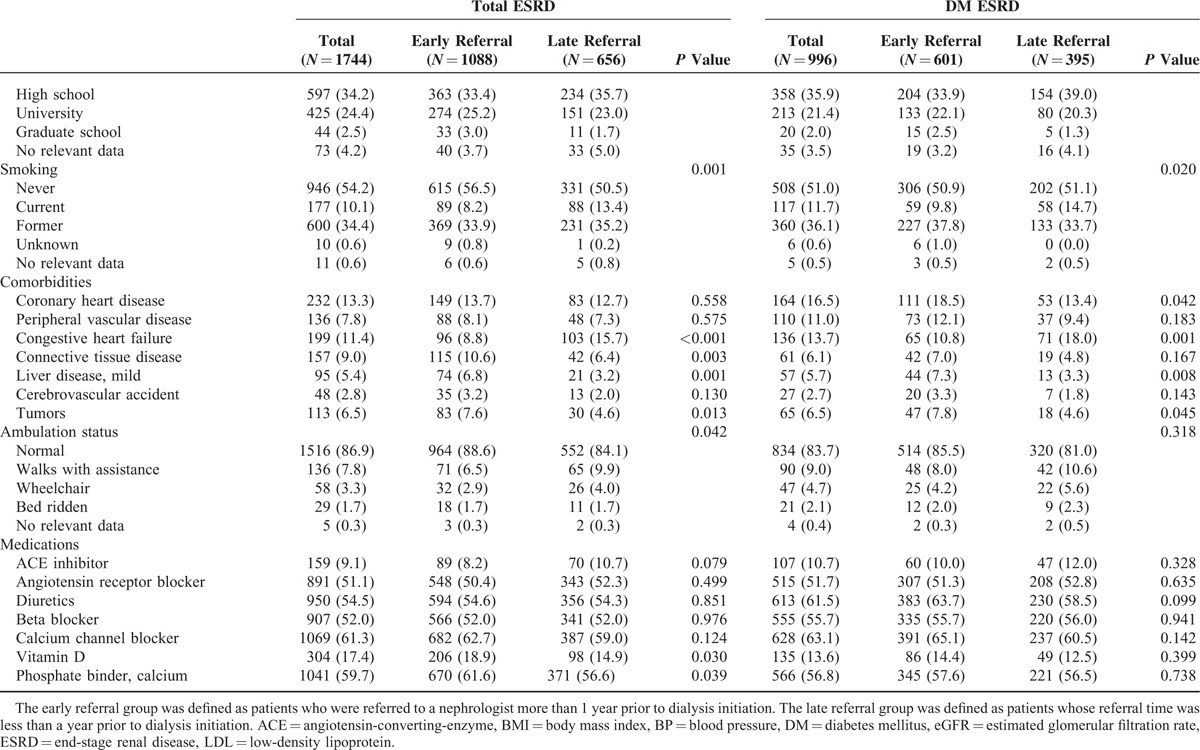
Patient Characteristics According to Referral Time

In the ER, the patients were younger at the time of referral to the nephrologists. At the referral time, the blood pressure, BUN, serum creatinine, and phosphorus levels were lower and the hemoglobin, albumin, calcium, total cholesterol level, and estimated glomerular filtration rate were higher in the ER than in the LR. In addition, more female patients were in the ER. According to the causes of primary renal disease, patients with glomerulonephritis were more common in the ER and patients with diabetes or hypertension were more common in the LR.

At the time of dialysis initiation, the patients’ ages in the ER were significantly higher compared with that of the LR (56.6 ± 13.7 vs 54.5 ± 14.2 year, *P* = 0.001). Although certain variables, including the systolic blood pressure, body mass index, uric acid, intact PTH, glucose, HbA1c, and total cholesterol levels were similar in both groups, the diastolic blood pressure (77.3 ± 13.9 vs 79.6 ± 14.5 mm Hg, *P* = 0.005), phosphorus levels (5.4 ± 1.8 vs 5.6 ± 2.0 mg/dL, *P* = 0.038), and LDL cholesterol levels (87.4 ± 38.5 vs 93.0 ± 37.3 mg/dL, *P* = 0.003) were lower, and the hemoglobin levels (9.0 ± 1.6 vs 8.9 ± 1.6 g/dL, *P* = 0.016) and calcium levels (7.9 ± 1.0 vs. 7.7 ± 1.1 mg/dL, *P* = 0.012) were higher in the ER.

The patients’ working status, medical insurance status, marriage status, familial numbers and support status, and social support status were not different between the ER and LR. However, in regard to occupation, professional specialist and office manager were more in the ER (*P* = 0.030). In addition, patients in the ER were more highly educated (university or graduate school) or uneducated (*P* = 0.050) and had a low proportion of current smokers (*P* = 0.001).

Regarding comorbidities, patients in the ER had a higher prevalence of connective tissue disease (*P* = 0.003), mild liver disease (*P* = 0.001), and tumors, including cancers (*P* = 0.013), whereas patients in the LR had a higher prevalence of congestive heart failure (*P* < 0.001). The modified Charlson comorbidity indexes were higher in the ER (5.3 ± 2.3 vs 5.0 ± 2.3, *P* = 0.014). Patients in the ER showed higher usage of vitamin D (*P* = 0.030) and calcium-based phosphate binder (*P* = 0.039).

Patients with diabetes mellitus as the cause of primary kidney disease presented similar characteristics as all of the ESRD patients between the ER and LR. Although the patients’ ages were similar between the 2 groups at referral, the patients were older at the start of dialysis in the ER (59.5. ± 12.0 vs 55.3 ± 12.7 year, *P* < 0.001). Patients in the ER had a higher modified Charlson comorbidity index (6.3 ± 2.1 vs 5.7 ± 2.2, *P* < 0.001) and a greater prevalence of coronary heart disease (*P* = 0.042), mild liver disease (*P* = 0.008), and tumors, including cancer (*P* = 0.045). However, the diastolic blood pressure (75.6 ± 13.4 vs 78.4 ± 14.4 mm Hg, *P* = 0.014) and LDL cholesterol levels (85.7 ± 40.7 vs 94.0 ± 39.5 mg/dL, *P* = 0.001) were lower in the ER. Patients in the LR had a higher prevalence of congestive heart failure (*P* = 0.001).

### Characteristics of Patients With Ultralate Referral

The characteristics of patients in the ULR (referral time <3 months) were compared with those of patients in the LR (referral time, 3–12 months) (Table 2 ). The patients’ age at the time of referral and time of dialysis initiation were not different between the groups. Patients in the ULR had a greater prevalence of hypertension as the cause of primary renal disease (*P* = 0.005). At the time of dialysis initiation, patients in the ULR presented lower modified Charlson comorbidity indexes (4.8 ± 2.2 vs 5.3 ± 2.3, *P* = 0.003). The social factors, including occupation, working status, insurance status, marriage status, familial or social support status, and education status, were not different between the ULR and LR. The prevalence of diuretics usage was significantly lower in the ULR (49.2% vs 62.0%, *P* = 0.002).

**TABLE 2:**
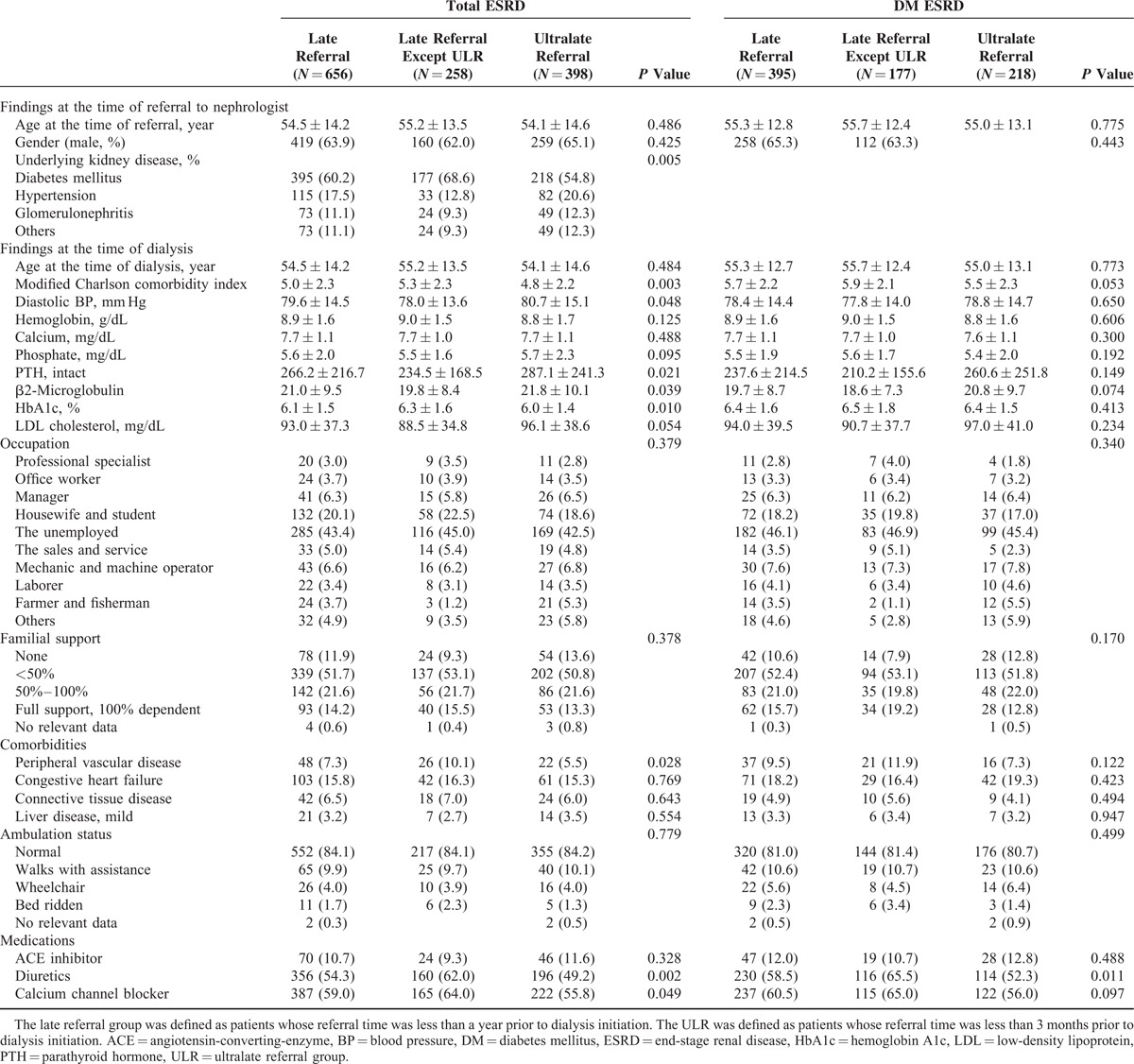
Characteristics of Patients in the Late Referral Group

### Demographic, Clinical, and Social Factors Affecting Referral Times

The demographic, clinical, and social factors were analyzed according to referral time (Table 3 ). Female patients were referred earlier than male patients (46.0 ± 59.4 vs 38.8 ± 51.6 months, *P* = 0.010), and patients with glomerulonephritis (63.3 ± 71.5 months) were referred earlier than those with hypertension (41.5 ± 55.0 months) or diabetes mellitus (31.3 ± 40.3 months, *P* < 0.001). Occupation (the sale and service, mechanic, laborer, and farmer or fisherman) was associated with short referral time (*P* = 0.028). A higher degree of familial support was associated with a long referral time (*P* = 0.001), and education status (highly educated, such as university or graduate school, or uneducated) was associated with a long referral time (*P* = 0.013). Current smoking was associated with a short referral time (28.7 ± 47.6 vs never smoking 45.8 ± 58.0, *P* < 0.001), and the use of vitamin D was associated with a long referral time (53.9 ± 64.3 vs 39.1 ± 52.5 months, *P* < 0.001). Among comorbidities, congestive heart disease (27.5 ± 40.4 vs 43.6 ± 56.3, *P* < 0.001) was associated with a short referral time, whereas connective tissue disease (62.0 ± 73.4 vs 39.7 ± 52.4 months, *P* < 0.001), mild liver disease (47.4 ± 46.7 vs 41.4 ± 55.4 months, *P* = 0.028), and neoplastic disease (54.7 ± 68.7 vs 40.8 ± 53.8 months, *P* = 0.012) were associated with a long referral time.

**TABLE 3:**
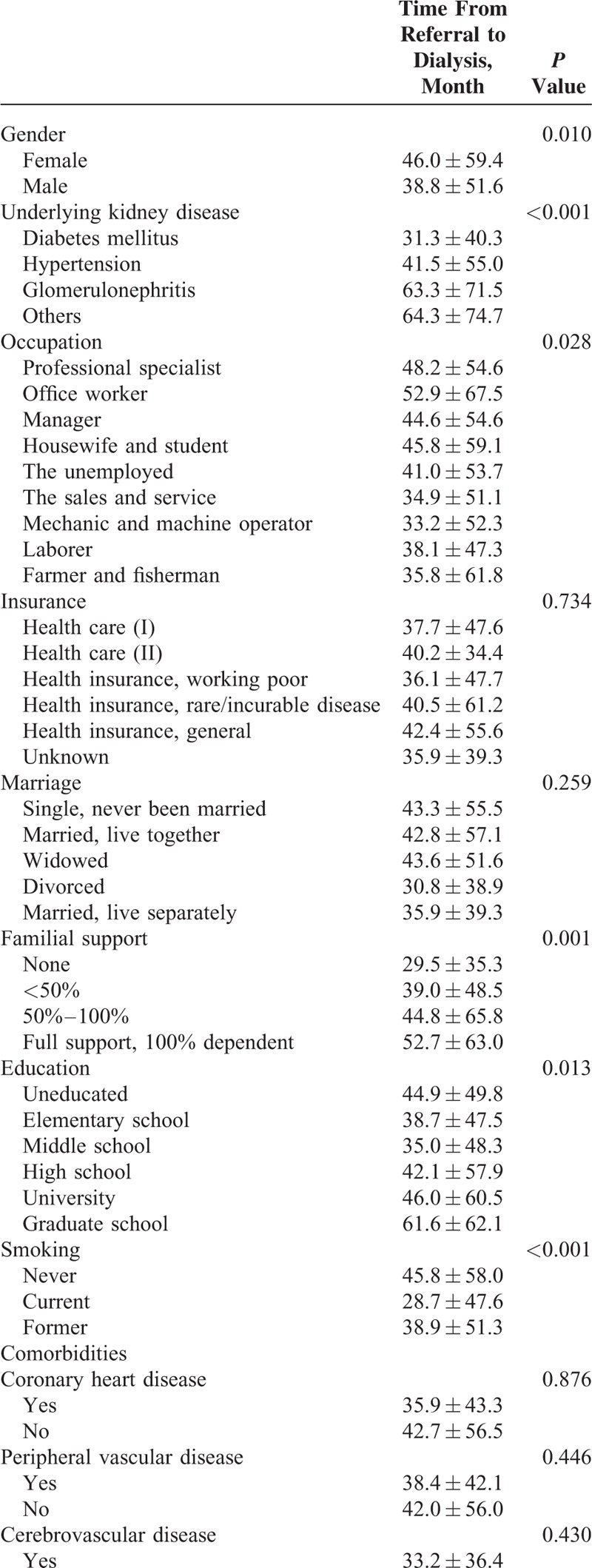
Analysis of Factors Affecting Referral Time

**TABLE 3 (Continued):**
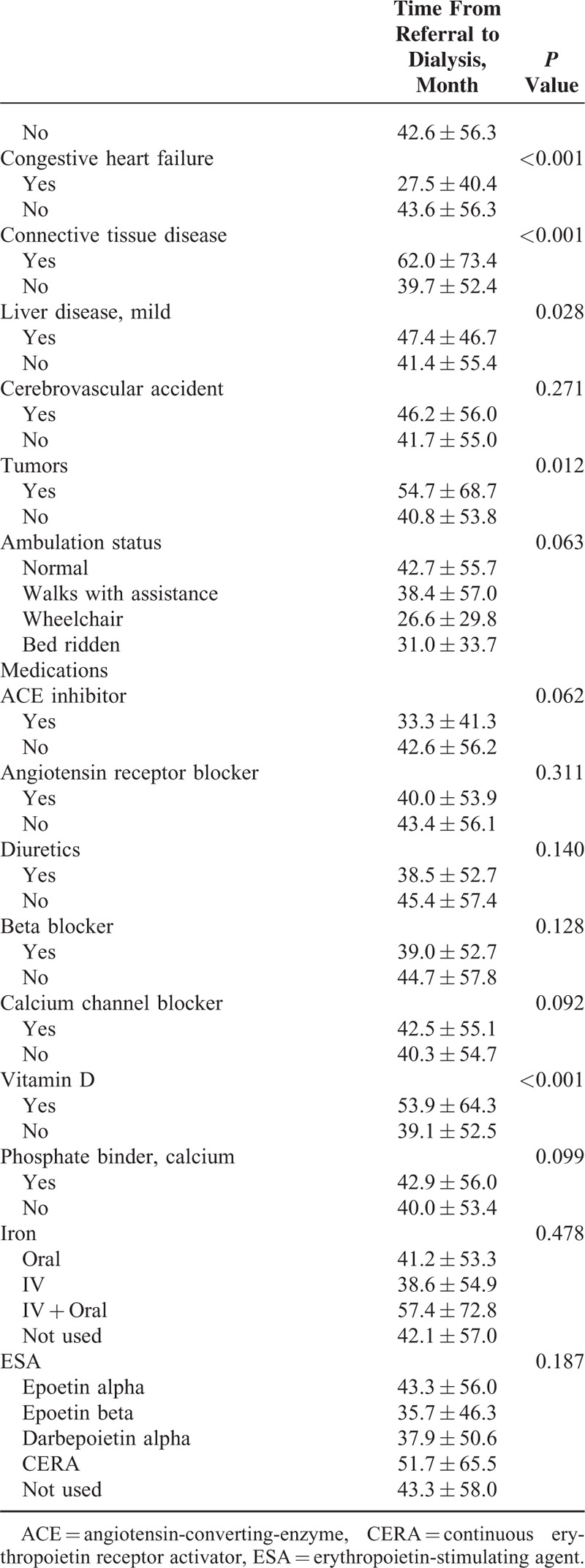
Analysis of Factors Affecting Referral Time

### Demographic, Clinical, and Social Factors Associated With Late and Ultralate Referral

The association of various demographic, clinical, and socio-economic factors with late and ultralate referral was analyzed with univariable logistic regression methods (Table 4). Male sex, underlying kidney disease (diabetes mellitus and hypertension), occupation (generally considered to be associated with low income and physical activity, including the sale and service, mechanic, laborer, and farmer or fisherman), smoking, congestive heart failure, and restricted ambulation with assistance were associated with late referral. Vitamin D and phosphate binder usage were associated with early referral in univariable analysis. Male sex, hypertensive kidney disease, occupation (mechanic, laborer, and farmer or fisherman), poor familial support, congestive heart failure, and not using diuretics were associated with ultralate referral. To validate the independent effects of various factors on late and ultralate referral, we analyzed the association using the multiple logistic regression methods (Table 5). Various factors, including male sex, hypertensive kidney disease, occupation, congestive heart failure, and restricted ambulation with assistance, were independently associated with late and ultralate referral. Diabetic kidney disease was associated with late referral, and smoking was associated with ultralate referral.

**TABLE 4:**
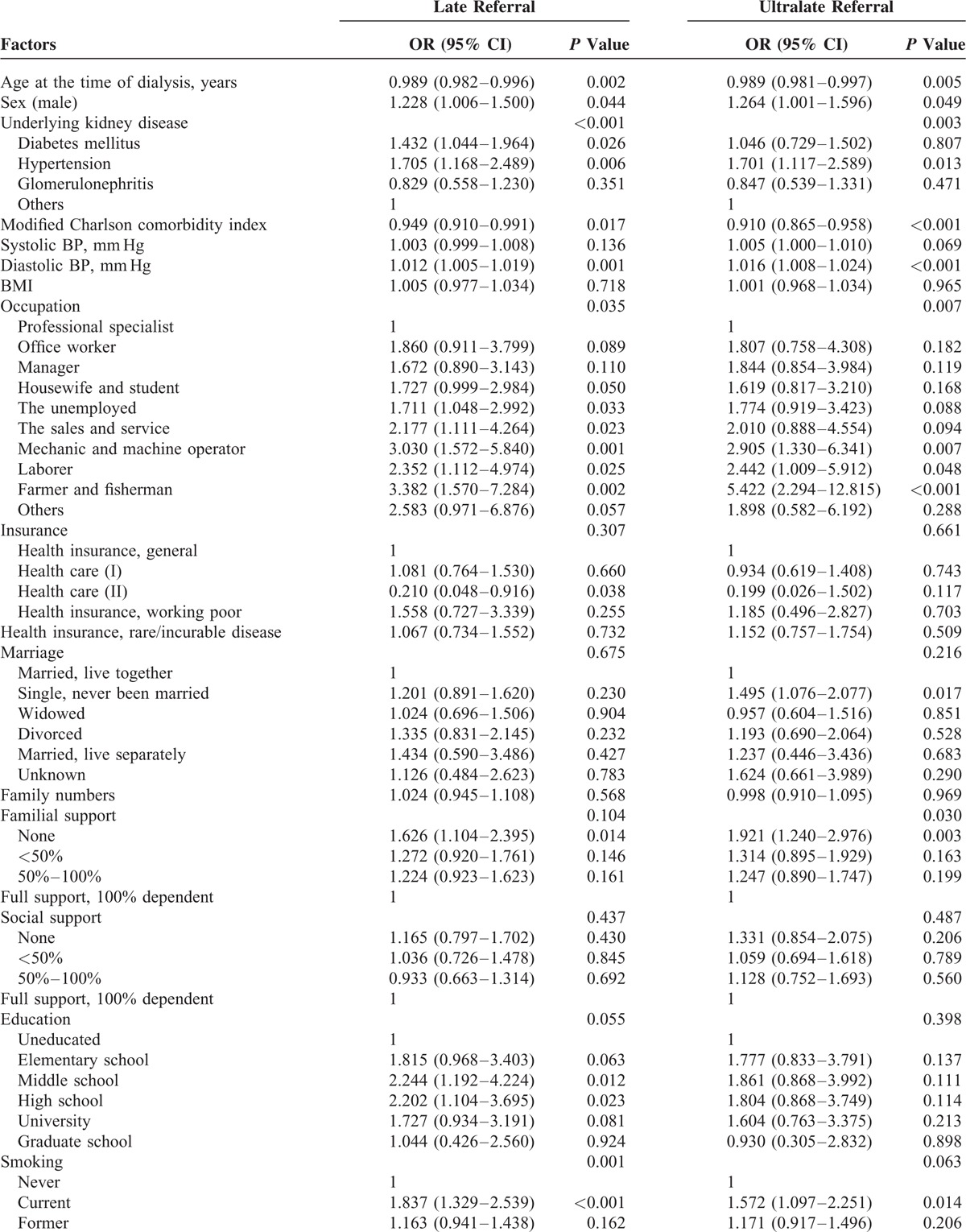
Univariable Analysis for Late and Ultralate Referral

**TABLE 4 (Continued):**
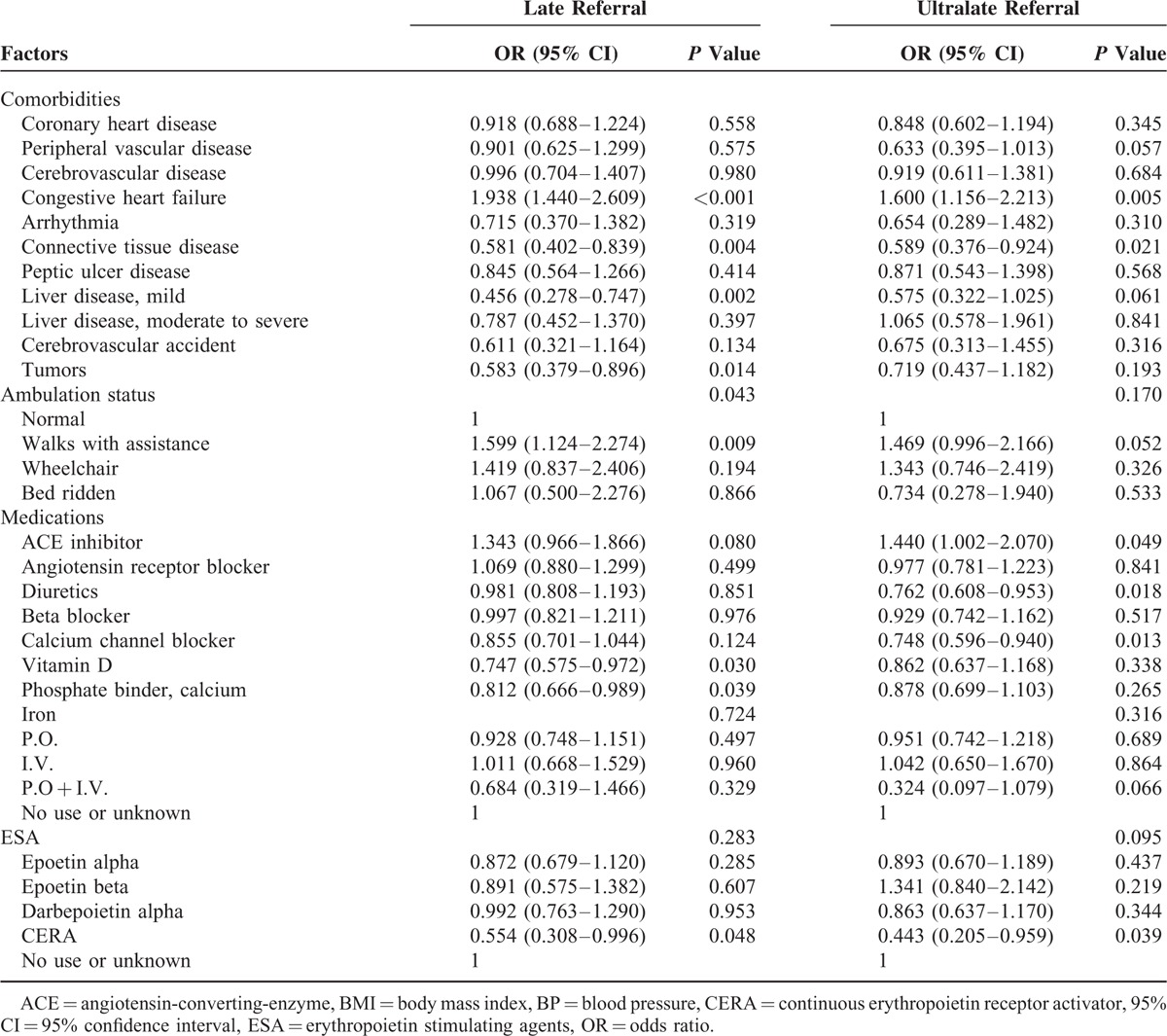
Univariable Analysis for Late and Ultralate Referral

**TABLE 5:**
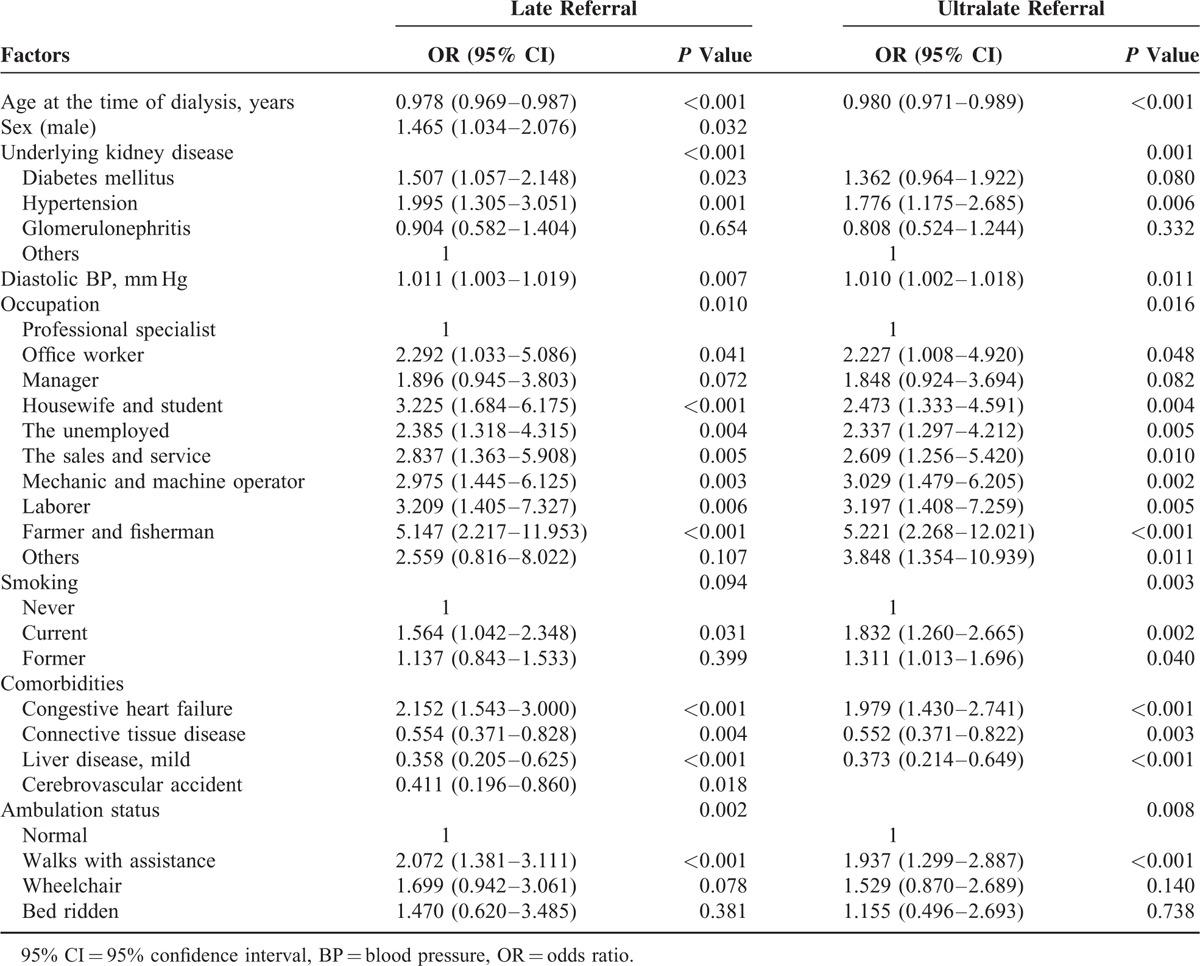
Multivariable Analysis for Late and Ultralate Referral

## DISCUSSION

We investigated the influence of various demographic, clinical, and social factors on the referral time from the initial visit to a nephrologist to the start of dialysis using a prospective cohort of patients with end-stage renal disease. We found that patients with hypertension or diabetes as a cause of kidney disease were referred later than those with glomerulonephritis. Hypertensive or diabetic kidney disease, occupation, congestive heart failure, and restricted ambulation with assistance were proved to be independently associated with late referral. In addition, patients who were female and presented connective tissue disease or neoplastic disease, a high degree of education or an uneducated status, and a high degree of familial support were associated with a long referral time, whereas patients who presented congestive heart failure and smoking habits were associated with a short referral time.

The causes of primary kidney disease are important factors that determine the referral time of CKD patients. Patients with glomerulonephritis are referred early throughout the world,^13–15^ which is easily understood because glomerulonephritis is treated almost exclusively in the nephrology department. The reason for the high proportion of kidney biopsies might be related to the high probability of glomerulonephritis in the ER. CKD patients with diabetes mellitus are recommended for early referral to prevent the rapid progression of kidney disease and improve patient survival.^16,17^ Many other studies have reported that patients with diabetic kidney disease are referred early at more than 3 or 4 month before dialysis initiation.^18–22^ Kessler et al^15^ and Kinchen et al^23^ reported that the most prevalent referral time for diabetic kidney disease patients is between 4 and 12 months. Contrary to expectations, the results of this study showed that patients with diabetes were referred late compared with patients with glomerulonephritis and polycystic kidney disease. When we compared the referral time according to the causes of primary kidney disease, the referral time for diabetic kidney disease (31.3 ± 40.3 months) was shorter than that for glomerulonephritis (63.3 ± 71.5 months) or hypertension (41.5 ± 55.0 months). Among the 996 patients with diabetic kidney disease, 395 (39.7%) patients were treated by their primary physician or endocrinologists and referred less than 12 months before dialysis initiation. These discrepancies result from the definition of an early or late referral. In this study, we defined an early referral as more than 12 months prior to the start of dialysis. The continuing problem of late referral for diabetic patients might result from the lack of knowledge on the benefits of early referral and optimum referral times among physicians.^24^ A referral time of 1 year is insufficient to adequately care for diabetic kidney disease patients or to prevent or delay the renal complications of diabetes mellitus. We showed that the clinical outcomes of patients with diabetic CKD can be associated with better clinical prognosis through early referral; lowering blood pressure, phosphorus levels, and LDL cholesterol levels; and start of dialysis at later time. Patients with diabetic kidney disease should be referred earlier at the stage of microalbuminuria or decreased kidney function (before CKD stage 3).

When we compared the comorbidities, the proportion of patients with congestive heart failure was high in the LR. Reduced kidney function and advanced CKD are high risk factors for heart failure.^25^ Volume overload, high blood pressure, increased renin–angiotensin–aldosterone system activity, and abnormal lipid metabolism, which are complicated in CKD, can contribute to the development of heart failure.^26^ Heart failure can aggravate the progression of CKD and increase the risk of end-stage renal disease and death in patients with CKD.^27,28^ Therefore, dietary salt restrictions and adequate blood pressure control, which are emphasized in the management of advanced CKD patients, are important for preventing volume overload and the development or aggravation of heart failure. Patients in the ER in this study could present a low prevalence of congestive heart failure because of adequate drug prescriptions and education regarding CKD and diet in the nephrology clinics. In fact, patients in the ER had low diastolic blood pressure and low cholesterol levels. Patients in the LR had a higher prevalence of congestive heart failure and a higher usage of diuretics, especially among patients who were referred between 3 and 12 months prior to starting dialysis. Patients in the ER are relatively well controlled with salt restriction, and patients in the LR might be treated less adequately for volume overload and high blood pressure. Therefore, the clinical need for diuretics would be increased in the LR.

Studies have suggested that demographic and socio-economic status affect the referral time to nephrologists and showed that discrepancies occur according to race, nations, and social or medical systems. In general, patients without insurance or employment and with low education and restricted ambulatory status are referred late.^20,23^ In this study, the status of medical insurance was consistent between the ER and LR. In South Korea, all patients have obligatory public medical insurance, and accessibility to medical facilities is relatively good. The status of medical insurance might affect referral time when the insurance system is diverse, nonobligatory, or not generalized to all populations. However, occupation, education, smoking, degree of familial support, and status of ambulation are important factors associated with referral time. Occupation (generally considered to be associated with low income and physical activity, including the sale and service, mechanician, laborer, and farmer or fisher) was associated with short referral time. Low level of education (elementary or middle school) was associated with short referral time, and highly educated patients who graduated from a university or finished graduate school and uneducated patients were strongly associated with long referral time. The association of uneducated patients with early referral might be related to hereditary or advanced kidney disease causing disturbances to normal education in childhood. Patients with reduced familial support and restricted ambulation are involuntarily susceptible to late referral. CKD patients with these socio-economic and lifestyle-related factors might experience difficulties or obstacles in visiting nephrology clinics. These results suggest the possibility of improving the status of nephrology referrals by a widespread social campaign or patient education to convey the importance of CKD and the reasons why patients should visit nephrologists at earlier stages.

The benefits of early referral to nephrologists are well known, and early referral improves patient survival and quality of life and reduces hospitalization, length of hospital stay, and medical costs.^1,29–32^ Using the CRC ESRD cohort in South Korea, we also demonstrated that early referral could provide benefits with regard to cardiovascular survival, medical expenses, and quality of life after dialysis initiation.^5–7^ In addition, this study showed that early referral can be associated with the delay of renal replacement therapy. Jones et al^33^ showed that reductions in the glomerular filtration rate slowed significantly after nephrology referral. These clinical benefits of early referral might arise from appropriate advanced CKD care during visits to nephrology clinics. Patients in the ER had visited nephrology clinics a greater number of times and had been educated regarding CKD and diet control to a greater degree compared with patients in the LR. The appropriate control of CKD in the ER resulted in improved patient health status at the start of dialysis, including a low diastolic blood pressure, high hemoglobin levels, low phosphorus levels, and low LDL cholesterol levels.

Herein, the association of various demographic, clinical, and social factors with referral time was analyzed using a large-scale prospective cohort study. However, because of the nature of cross-sectional studies, we cannot directly prove the causal relationship between various factors and referral time. The results of this study, that revealed the factors associated with early or late referral to nephrologists, can be used in clinical practice to improve the care of patients with CKD and increase the proportion of patients who are referred early to nephrologists. In future studies, it will be helpful to investigate the optimal time of referral that is sufficient to delay the progression of kidney disease and prevent the episodes of heart failure aggravation, among CKD patients with diabetes mellitus or heart failure.

In conclusion, despite the various benefits of early referral, a large proportion of CKD patients with hypertension or diabetes are referred later than those with glomerulonephritis. Male sex, diabetic or hypertensive kidney disease, occupation, congestive heart failure, and restricted ambulation with assistance are independent risk factors for late referral. A low level of education, low familial support status, and smoking habits are factors associated with short referral time. Education regarding CKD and the benefits of early referral might be helpful to increase the number of patients who are referred early. Clinicians should be aware of the factors associated with late referral and attempt to refer these patients at an earlier CKD stage to improve clinical outcomes.

## Supplementary Material

Supplemental Digital Content
