# Supplementary material for: Factors Affecting the Referral Time to Nephrologists in Patients With Chronic Kidney Disease: A Prospective Cohort Study in Korea
Source: Medicine (Baltimore). 2016 May 13;95(19):e3648. doi: 10.1097/MD.0000000000003648 (PMC4902530; doi:10.1097/MD.0000000000003648)
Supplement: Supplemental Digital Content [file medi-95-e3648-s001.doc]

**Supplementary Table 1.** Patient characteristics according to referral time

|  | Total ESRD |  |  |  | DM ESRD |  |  |  |
| --- | --- | --- | --- | --- | --- | --- | --- | --- |
|  | Total  (*N* =1744) | Early referral  (*N* =1088) | Late referral  (*N* =656) | *P* value | Total  (*N* =996) | Early referral  (*N* =601) | Late referral  (*N* =395) | *P* value |
| Findings at the time of referral to nephrologist |  |  |  |  |  |  |  |  |
| BUN (mg/dL) | 51.2 ± 34.3 | 36.6 ± 20.1 | 71.2 ± 39.3 | < 0.001 | 48.8 ± 30.5 | 36.0 ± 17.5 | 65.5 ± 35.3 | < 0.001 |
| Calcium (mg/dL) | 8.2 ± 1.2 | 8.6 ± 1.1 | 7.8 ± 1.1 | < 0.001 | 8.2 ± 1.1 | 8.6 ± 1.0 | 7.8 ± 1.1 | < 0.001 |
| Phosphorus (mg/dL) | 4.7 ± 1.7 | 4.1 ± 1.0 | 5.5 ± 2.0 | < 0.001 | 4.6 ± 1.4 | 4.1 ± 0.8 | 5.3 ± 1.6 | < 0.001 |
| Total cholesterol (mg/dL) | 188.4 ± 62.9 | 194.0 ± 63.7 | 180.8 ± 61.1 | < 0.001 | 192.0 ± 65.3 | 195.6 ± 65.0 | 187.1 ± 65.5 | 0.046 |
| Number of visits to a nephrologist from referral to dialysis (%) |  |  |  | <0.001 |  |  |  | <0.001 |
| None | 27 (1.5) | 2 (0.2) | 25 (3.8) |  | 13 (1.3) | 2 (0.3) | 11 (2.8) |  |
| 1 time | 176(10.1) | 8 (0.7) | 168 (25.6) |  | 104 (10.4) | 6 (1.0) | 98 (24.8) |  |
| 2 times or more | 1521 (87.2) | 1067 (98.1) | 454 (69.2) |  | 869 (87.2) | 587 (97.7) | 282 (71.4) |  |
| Unknown | 17 (1.0) | 10 (0.9) | 7 (1.1) |  | 9 (0.9) | 5 (0.8) | 4 (1.0) |  |
| No relevant data | 3 (0.2) | 1 (0.1) | 2 (0.3) |  | 1 (0.1) | 1 (0.2) | 0 (0.0) |  |
| Education for dialysis | 1580 (90.6) | 1000 (91.9) | 580 (88.4) | 0.030 | 908 (91.2) | 554 (92.2) | 354 (89.6) | 0.039 |
| Diet education for CKD/ESRD | 1577 (89.3) | 987 (90.7) | 570 (86.9) | 0.046 | 892 (89.6) | 545 (90.7) | 347 (87.8) | 0.176 |
|  |  |  |  |  |  |  |  |  |
| Kidney biopsy | 290 (16.6) | 214 (19.7) | 76 (11.6) | < 0.001 | 84 (8.4%) | 54 (9.1) | 30 (7.7) | 0.641 |
| Dialysis (initial) |  |  |  | 0.652 |  |  |  | 0.409 |
| Hemodialysis | 1177 (67.5) | 730 (67.1) | 447 (68.1) |  | 708 (71.1) | 433 (72.0) | 275 (69.6) |  |
| Peritoneal dialysis | 567 (32.5) | 358 (32.9) | 209 (31.9) |  | 288 (28.9) | 168 (28.0) | 120 (30.4) |  |
| Dialysis (3 month, 2^nd^ visit) |  |  |  | 0.598 |  |  |  | 0.321 |
| Hemodialysis | 1151 (66.0) | 713 (65.5) | 438 (66.8) |  | 696 (69.9) | 427 (71.0) | 269 (68.1) |  |
| Peritoneal dialysis | 593 (34.0) | 375 (34.5) | 218 (33.2) |  | 300 (30.1) | 174 (29.0) | 126 (31.9) |  |
| Dialysis mode change | 102 (5.8) | 63 (5.8) | 39 (5.9) | 0.894 | 53 (5.3) | 34 (5.7) | 19 (4.8) | 0.560 |
|  |  |  |  |  |  |  |  |  |
| Findings at the time of dialysis |  |  |  |  |  |  |  |  |
| Uric acid (mg/dL) | 8.0 ± 2.6 | 8.0 ± 2.6 | 8.1 ± 2.5 | 0.111 | 7.9 ± 2.4 | 7.9 ± 2.4 | 8.0 ± 2.3 | 0.359 |
| PTH, intact | 269.2 ± 239.6 | 271.1 ± 252.6 | 266.2 ± 216.7 | 0.671 | 221.1 ± 177.6 | 210.2 ± 147.6 | 237.6 ± 214.5 | 0.369 |
| Glucose (mg/dL) | 139.2 ± 72.6 | 139.2 ± 72.5 | 139.1 ± 72.8 | 0.753 | 156.5 ± 83.9 | 157.8 ± 84.5 | 154.6 ± 83.0 | 0.749 |
| HbA1c (%) | 6.1 ± 1.6 | 6.1 ± 1.6 | 6.1 ± 1.5 | 0.990 | 6.5 ± 1.7 | 6.6 ± 1.8 | 6.4 ± 1.6 | 0.062 |
| Total cholesterol (mg/dL) | 157.2 ± 48.4 | 155.8 ± 49.1 | 159.6 ± 47.2 | 0.080 | 156.4 ± 51.9 | 153.6 ± 51.7 | 160.6 ± 52.1 | 0.044 |
| TSAT | 36.1 ± 67.3 | 34.7 ± 50.8 | 38.3 ± 87.6 | 0.865 | 35.3 ± 75.6 | 32.9 ± 38.7 | 38.8 ± 108.8 | 0.446 |
| Insurance |  |  |  | 0.221 |  |  |  | 0.186 |
| Health care (I) | 148 (8.5) | 90 (8.3) | 58 (8.8) |  | 98 (9.8) | 52 (8.7) | 46 (11.6) |  |
| Health care (II) | 18 (1.0) | 16 (1.5) | 2 (0.3) |  | 13 (1.3) | 11 (1.8) | 2 (0.5) |  |
| Health insurance, working poor | 27 (1.5) | 14 (1.3) | 13 (2.0) |  | 21 (2.1) | 10 (1.7) | 11 (2.8) |  |
| Health insurance, rare/incurable disease | 126 (7.2) | 77 (7.1) | 49 (7.5) |  | 74 (7.4) | 44 (7.3) | 30 (7.6) |  |
| Health insurance, general | 1403 (80.4) | 879 (80.8) | 524 (79.9) |  | 775 (77.8) | 476 (79.2) | 299 (75.7) |  |
| Unknown | 15 (0.9) | 8 (0.7) | 7 (1.1) |  | 11 (1.1) | 5 (0.8) | 6 (1.5) |  |
| No relevant data | 7 (0.4) | 4 (0.4) | 1 (0.2) |  | 4 (1.1) | 3 (0.5) | 1 (0.1) |  |
| Marriage |  |  |  | 0.673 |  |  |  | 0.251 |
| Single, never been married | 209 (12.0) | 124 (11.4) | 85 (13.0) |  | 80 (8.0) | 39 (6.5) | 41 (10.4) |  |
| Married, live together | 1244 (71.3) | 792 (72.8) | 452 (68.9) |  | 750 (75.3) | 467 (77.7) | 283 (71.6) |  |
| Widowed | 122 (7.0) | 77 (7.1) | 45 (6.9) |  | 61 (6.1) | 35 (5.8) | 26 (6.6) |  |
| Divorced | 74 (4.2) | 42 (3.9) | 32 (4.9) |  | 51 (5.1) | 29 (4.8) | 22 (5.6) |  |
| Married, live separately | 20 (1.1) | 11 (1.0) | 9 (1.4) |  | 14 (1.4) | 8 (1.3) | 6 (1.5) |  |
| Unknown | 23 (1.3) | 14 (1.3) | 9 (1.4) |  | 13 (1.3) | 9 (1.5) | 4 (1.0) |  |
| No relevant data | 52 (3.0) | 28 (2.6) | 24 (3.7) |  | 27 (2.7) | 14 (2.3) | 13 (3.3) |  |
| Family numbers | 2.9 ± 1.3 | 2.9 ± 1.3 | 3.0 ± 1.3 | 0.578 | 2.9 ± 1.3 | 2.9 ± 1.3 | 3.0 ± 1.3 | 0.395 |
| Familial support |  |  |  | 0.102 |  |  |  | 0.927 |
| None | 176 (10.1) | 98 (9.0) | 78 (11.9) |  | 98 (9.8) | 56 (9.3) | 42 (10.6) |  |
| < 50% | 905 (51.9) | 566 (52.0) | 339 (51.7) |  | 528 (53.0) | 321 (53.4) | 207 (52.4) |  |
| 50-100% | 370 (21.2) | 228 (21.0) | 142 (21.6) |  | 209 (21.0) | 126 (21.0) | 83 (21.0) |  |
| Full support, 100% dependent | 283 (16.2) | 190 (17.5) | 93 (14.2) |  | 157 (15.8) | 95 (15.8) | 62 (15.7) |  |
| No relevant data | 10 (0.6) | 6 (0.6) | 4 (0.6) |  | 4 (0.4) | 3 (0.5) | 1 (0.3) |  |
| Social support |  |  |  | 0.437 |  |  |  | 0.880 |
| None | 322 (18.5) | 190 (17.5) | 132 (20.1) |  | 166 (16.7) | 98 (16.3) | 68 (17.2) |  |
| < 50% | 730 (41.9) | 469 (43.1) | 261 (39.8) |  | 421 (42.3) | 260 (43.3) | 161 (40.8) |  |
| 50-100% | 508 (29.1) | 314 (28.9) | 194 (29.6) |  | 300 (30.1) | 178 (29.6) | 122 (30.9) |  |
| Full support, 100% dependent | 174 (10.0) | 109 (10.0) | 65 (9.9) |  | 105 (10.5) | 62 (10.3) | 43 (10.9) |  |
| No relevant data | 10 (0.6) | 6 (0.6) | 4 (0.6) |  | 4 (0.4) | 3 (0.5) | 1 (0.3) |  |
| Comorbidities |  |  |  |  |  |  |  |  |
| Cerebrovascular disease | 150 (8.6) | 94 (8.6) | 56 (8.5) | 0.980 | 89 (8.9) | 54 (9.0) | 35 (8.9) | 0.998 |
| Arrhythmia | 43 (2.5) | 30 (2.8) | 13 (2.0) | 0.317 | 25 (2.5) | 19 (3.2) | 6 (1.5) | 0.108 |
| Peptic ulcer disease | 112 (6.4) | 74 (6.8) | 38 (5.8) | 0.414 | 69 (6.9) | 43 (7.2) | 26 (6.6) | 0.752 |
| Liver disease, moderate to severe | 59 (3.4) | 40 (3.7) | 19 (2.9) | 0.396 | 37 (3.7) | 22 (3.7) | 15 (3.8) | 0.888 |
| Ambulation status |  |  |  | 0.042 |  |  |  | 0.318 |
| Normal | 1516 (86.9) | 964 (88.6) | 552 (84.1) |  | 834 (83.7) | 514 (85.5) | 320 (81.0) |  |
| Walks with assistance | 136 (7.8) | 71 (6.5) | 65 (9.9) |  | 90 (9.0) | 48 (8.0) | 42 (10.6) |  |
| Wheelchair | 58 (3.3) | 32 (2.9) | 26 (4.0) |  | 47 (4.7) | 25 (4.2) | 22 (5.6) |  |
| Bed ridden | 29 (1.7) | 18 (1.7) | 11 (1.7) |  | 21 (2.1) | 12 (2.0) | 9 (2.3) |  |
| No relevant data | 5 (0.3) | 3 (0.3) | 2 (0.3) |  | 4 (0.4) | 2 (0.3) | 2 (0.5) |  |
| Medications |  |  |  |  |  |  |  |  |
| Iron |  |  |  | 0.683 |  |  |  | 0.836 |
| Oral | 1075 (61.6) | 676 (62.1) | 399 (60.8) |  | 616 (61.8) | 375 (62.4) | 241 (61.0) |  |
| IV | 115 (6.6) | 70 (6.4) | 45 (6.9) |  | 72 (7.2) | 41 (6.8) | 31 (7.8) |  |
| IV + Oral | 33 (1.9) | 23 (2.1) | 10 (1.5) |  | 21 (2.1) | 12 (2.0) | 9 (2.3) |  |
| Not used | 512 (29.4) | 314 (28.9) | 198 (30.2) |  | 283 (28.4) | 172 (28.6) | 111 (28.1) |  |
| Unknown | 5 (0.3) | 2 (0.2) | 3 (0.5) |  | 3 (0.3) | 1 (0.2) | 2 (0.5) |  |
| No relevant data | 4 (0.2) | 3 (0.3) | 1 (0.2) |  | 1 (0.1) |  | 1 (0.3) |  |
| ESA |  |  |  | 0.400 |  |  |  | 0.688 |
| Epoetin alpha | 631 (36.2) | 402 (36.9) | 229 (34.9) |  | 378 (38.0) | 229 (38.1) | 149 (37.7) |  |
| Epoetin beta | 105 (6.1) | 67 (6.2) | 39 (5.9) |  | 57 (5.7) | 36 (6.0) | 21 (5.3) |  |
| Darbepoietin alpha | 496 (28.4) | 301 (27.7) | 195 (29.7) |  | 286 (28.7) | 177 (29.5) | 109 (27.6) |  |
| CERA | 64 (3.7) | 47 (4.3) | 17 (2.6) |  | 31 (3.1) | 21 (3.5) | 10 (2.5) |  |
| Not used | 435 (24.9) | 263 (24.2) | 172 (26.2) |  | 238 (23.9) | 136 (22.6) | 102 (25.8) |  |
| Unknown | 8 (0.5) | 5 (0.5) | 3 (0.5) |  | 5 (0.5) | 2 (0.3) | 3 (0.8) |  |
| No relevant data | 4 (0.2) | 3 (0.3) | 1 (0.2) |  | 1 (0.1) |  | 1 (0.3) |  |

The early referral group was defined as patients who were referred to a nephrologist more than 1 year prior to dialysis initiation. The late referral group was defined as patients whose referral time was less than a year prior to dialysis initiation.

BUN, blood urea nitrogen; CERA, continuous erythropoietin receptor activator; CKD, chronic kidney disease; ESRD, end-stage renal disease
